# Interface Acoustic Waves in 128° YX-LiNbO_3_/SU-8/Overcoat Structures

**DOI:** 10.3390/mi16010099

**Published:** 2025-01-16

**Authors:** Cinzia Caliendo, Massimiliano Benetti, Domenico Cannatà, Farouk Laidoudi, Gaetana Petrone

**Affiliations:** 1 Institute for Photonics and Nanotechnologies, IFN-CNR, Via del Fosso del Cavaliere 100, 00133 Rome, Italy; 2 Institute for Microelectronics and Microsystems, IMM-CNR, Via del Fosso del Cavaliere 100, 00133 Rome, Italy; massimiliano.benetti@cnr.it (M.B.); domenico.cannata@cnr.it (D.C.); 3 Research Center in Industrial Technologies CRTI, P.O. Box 64 Cheraga, Algiers 16014, Algeria; f.laidoudi19@gmail.com; 4 Department of Astronautical, Electrical and Energy Engineering, University of Rome “La Sapienza”, Via Eudossiana 18, 00184 Rome, Italy; gaetana.petrone@artov.imm.cnr.it

**Keywords:** IAW, piezoelectricity, LiNbO_3_, fused silica, silicon, Al_2_O_3_

## Abstract

The propagation of interface acoustic waves (IAWs) in 128° YX-LiNbO_3_/SU-8/overcoat structures was theoretically studied and experimentally investigated for different types of overcoat materials and thicknesses of the SU-8 adhesive layer. Three-dimensional finite element method analysis was performed using Comsol Multiphysics software to design an optimized multilayer configuration able to achieve an efficient guiding effect of the IAW at the LiNbO_3_/overcoat interface. Numerical analysis results showed the following: (i) an overcoat faster than the piezoelectric half-space ensures that the wave propagation is confined mainly close to the surface of the LiNbO_3_, although with minimal scattering in the overcoat; (ii) the presence of the SU-8, in addition to performing the essential function of an adhesive layer, can also promote the trapping of the acoustic energy toward the surface of the piezoelectric substrate; and (iii) the electromechanical coupling efficiency of the IAW is very close to that of the surface acoustic wave (SAW) along the bare LiNbO_3_ half-space. The numerical predictions were experimentally assessed for some SU-8 layer thicknesses and overcoat material types. The propagation of the IAWs was experimentally measured in LiNbO_3_/SU-8/fused silica, LiNbO_3_/SU-8/(001)Si, and LiNbO_3_/SU-8/c-Al_2_O_3_ structures for an SU-8 layer about 15 µm thick; the velocities of the IAWs were found in good agreement with the theoretically calculated values. Although the interest in IAWs was born many years ago for packageless applications, it can currently be renewed if thought for applications in microfluidics. Indeed, the IAWs may represent a valid alternative to standing SAWs, which are strongly attenuated when travelling beneath the walls of polydimethylsiloxane (PDMS) microfluidic channels for continuous flow particle manipulation, provided that the channel is excavated into the overcoating.

## 1. Introduction

Interface acoustic waves (IAWs), also known as Stoneley waves, propagate along the boundary between two perfectly bonded solids. Their propagation is allowed under the condition that the velocity of the slow shear bulk acoustic wave (BAW) of the overcoat material is faster than the velocity of the slow shear BAW of the substrate. The IAW is non-dispersive since the propagating medium has no characteristic dimensions; its velocity lies between the velocity of the free SAW and that of the slowest BAW velocity [1], and its displacement amplitude decays to zero within a few wavelengths away from either side of the interface.

The interest around IAWs exploded about few decades ago for the design of package-less devices [2,3,4] thanks to the numerous advantages they offered compared to conventional SAW-based devices, such as radio frequency filters and resonators, which find application in fields like aerospace, communication, automotive, etc., to cite just a few. Despite their small size, these devices must be encapsulated inside a package that must be hermetic to protect them from environmental disturbances (such as dust and humidity, radiation environment or simply variations in ambient pressure and temperature) and high enough to avoid contact between the surface of the SAW device and the package itself; consequently, they are expensive and represent an obstacle to the miniaturization of the devices. The electroacoustic devices based on the propagation of IAWs are advantageous for many reasons: (1) the cavity formation is not required since the wave energy is concentrated at the interface between two bulk media; (2) they are smaller than their SAW-based counterparts since do not necessitate the presence of a cavity; (3) their manufacturing process is similar to that of conventional SAW filters or resonators; (4) the IAWs can be excited and received by interdigital transducers (IDTs). In Reference [5], two types of IAWs are studied in the SiO_2_/Y-rotated X-propagation LiNbO_3_ structure: one wave exhibits a shear horizontal polarization (SH-IAW) and the other an elliptical polarization (Stonely wave). Depending on the LiNbO_3_ y-axis rotation angle μ, the electromechanical coupling coefficient K^2^ of the SH-IAW can be as high as 16.7% while that of the Stoneley wave reaches 5%; moreover, the propagation of the Stonely or of the SH-IAW can be suppressed for a proper choice of µ.

SAW-driven microfluidic devices [6,7,8] typically use Rayleigh type SAWs on 128° Y LiNbO_3_ substrates, which have a strong K^2^ and large mechanical displacement amplitudes. Notably, the K^2^ for 128° YX LiNbO_3_ (5.3%) is much higher than that for the Rayleigh waves generated in quartz (0.16%), ZnO (1.0%), X-LiTaO_3_ (1.12%), or c-AlN (0.4%). The polydimethylsiloxane (PDMS) channel technology in combination with LiNbO_3_ substrates has been proven to be an efficient mechanism to couple the SAW energy into the liquid flowing inside the channel. A multifunctional microfluidic platform can be fabricated in a monolithic piezoelectric substrate where PDMS channels (with side and top walls) are fabricated: the liquid to be tested flows inside the channel with dispersed particles that migrate towards the pressure nodes when subjected to the acoustic radiation forces induced by the stationary SAWs; such devices find application in many biomedical and chemical fields to support fluid mixing, particle manipulations, as well as the detection of various biological particles [9]. However, the SAW is highly attenuated when travelling under the walls of the PDMS microchannels, which absorb the acoustic energy, thus limiting the amount of energy coupled inside the microfluidic channel.

The deficiencies of the SAW-based systems can be alleviated by performing particle manipulation based on IAWs, as reported in Reference [10]; here, for the first time, the propagation of the Stoneley wave in a ZX-LiTaO_3_/SU-8(5 μm)/fused silica structure is experimentally assessed. In Reference [11], the use of IAWs in the Z-LiTaO_3_/SU-8/SiO_2_ structure was proposed for continuous flow submicron particle manipulation and particularly for the sorting of biologically interesting samples such as bacteria, viruses, DNA, etc. In Reference [11], the authors demonstrated that IAW-based devices can be used to manipulate particles as small as 0.5 μm in diameter.

The numerical analysis performed in Reference [12] demonstrates that the out-of-plane displacement component of the SAWs propagating along the surface of 128° YX-LiNbO_3_ have amplitudes quite higher than that along other LiNbO_3_ cuts; moreover, the 128° YX-LiNbO_3_ offers the advantage to have quite high electroacoustic coupling coefficient. These characteristics make the 128° YX-LiNbO_3_ a piezoelectric substrate interesting to test for IAW-application-based acoustofluidics. To successfully excite the propagation of IAWs, both the overcoat and the LiNbO_3_ substrate need to be bonded together by using, for example, a thin intermediate adhesive layer such as a thin spin-coated SU-8 film. The thickness of SU-8 needs to be carefully selected to avoid unwanted IAW energy leakage effects and to ensure, at the same time, a fairly good electromechanical coupling coefficient.

The present paper aims to demonstrate that the 128° YX-LiNbO_3_, if coupled with specific overcoat materials, can sustain the propagation of IAWs, showing properties that can be exploited for microfluidic application, such as a large shear vertical displacement component and an electroacoustic coupling efficiency (about 4.8%) very close to that of the SAW in bare 128° YX-LiNbO_3_ (5.2%). The characteristics of IAWs propagating in multilayered structures composed of a 128° YX-LiNbO_3_ substrate and an overcoat material bonded together by an SU-8 adhesive layer were theoretically investigated for different overcoat materials and SU-8 layer thicknesses with respect to the phase velocity, propagation loss, electromechanical coupling efficiency, and vertical displacement component. The propagation of the IAWs was experimentally assessed in LiNbO_3_/SU-8/fused silica, LiNbO_3_/SU-8/(001)Si, and LiNbO_3_/SU-8/c-Al_2_O_3_ structures; the velocities of the IAWs were found in good agreement with the theoretically calculated values.

## 2. Methods

The characteristics of the propagation of IAWs in multilayered structures composed of a 128° YX-LiNbO_3_ and an overcoat material bonded together by means of an SU-8 adhesive layer have been studied in terms of wave propagation loss and phase velocity, electromechanical coupling efficiency, and vertical displacement components for different overcoat materials and SU-8 layer thicknesses. Optimized designs have been found, which consist of specific types of overcoat material and thicknesses of the SU-8 layer. Then, the propagation of the IAWs was experimentally assessed in LiNbO_3_/SU-8/fused silica, LiNbO_3_/SU-8/(001)Si, and LiNbO_3_/SU-8/c-Al_2_O_3_ structures.

Three-dimension finite element method (FEM) modal analysis was performed by using Comsol Multiphysics 6.1 software. The model uses the piezoelectric Multiphysics coupling node comprising solid mechanics and electrostatic interfaces. The 128° YX crystal cut was obtained by the application of a rotated coordinate system based on the Euler angles of (0°, 38°, 0°). A unit cell was assumed to study the propagation of the IAWs. It consists of three domains: the LiNbO_3_ substrate (1000 µm thick), the SU-8 layer (with variable thickness), and the overcoat (500 µm thick). Periodic boundary conditions were applied at the lateral sides of the unit cell (width λ and depth λ/4), parallel to x and y directions; free and fixed conditions were applied to the top and bottom surface of the structure. Some overcoat materials were studied, which show elastic properties quite different from those of the LiNbO_3_. An extremely fine mesh was chosen for all the FEM simulations to obtain more accurate results. Table 1 lists the elastic properties of the overcoat materials that were investigated.

### Eigenfrequency and Frequency Domain Study

**Figure 1:**
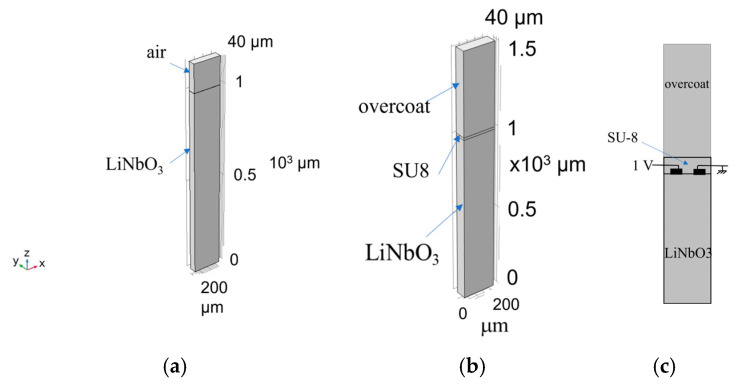
(**a**) The LiNbO_3_/air-based unit cell. (**b**) The LiNbO_3_/IDT/SU-8/overcoat-based unit cell. (**c**) The cross section (not in scale) of the LiNbO_3_/IDT/SU-8/overcoat-based unit cell, showing the two Al electrodes.

An eigenfrequency study was performed to calculate the natural frequencies of the propagating mode. First, we considered a unit cell including only a bare 128° YX-LiNbO_3_ half-space and an air domain (as shown in Figure 1a) to obtain the SAW’ eigenmode; then, an SU-8 layer and an overcoat were added to evaluate the IAW eigenmode (Figure 1b).

The frequency domain study was performed to calculate the admittance Y vs. the frequency curve at different SU-8 layer thicknesses and types of overcoat materials; two Al electrodes (width λ/4 and 150 nm thickness) were located onto the LiNbO_3_ surface (one electrode is grounded, the other is connected to a fixed electric potential of 1 V). Figure 1 c shows the cross section (not in scale) of the LiNbO_3_/IDT/SU-8/overcoat-based unit cell. The same damping (isotropic loss factor) of 10^−4^ was attributed to the LiNbO_3_ half-space (with also permittivity loss factor of 10^−4^) as well as to the SU-8 and overcoat materials.

**Figure 2:**
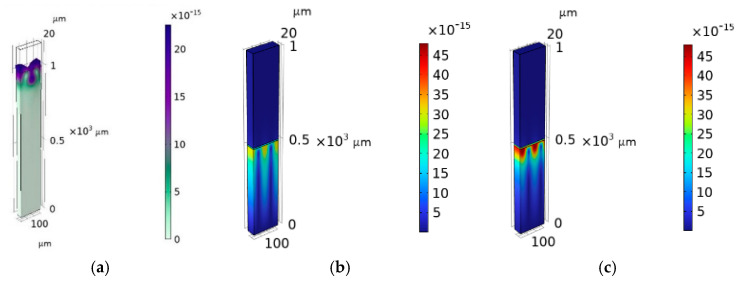
The solid displacement of (**a**) the Rayleigh wave in the LiNbO_3_/Air domain; (**b**) the IAW travelling in LiNbO_3_/SU-8/Al_2_O_3_ for an SU-8 layer 8 µm thick; and (**c**) the IAW travelling in LiNbO_3_/SU-8/Al_2_O_3_ for an SU-8 layer 10 µm thick.

A straight-crested non-dispersive Rayleigh wave travels along the surface of the bare piezoelectric substrate: the wave has three particle displacement components (the shear horizontal component is much shorter that the shear vertical and longitudinal one) and travels at a velocity of about 3994.80 m/s. The solid displacement of the Rayleigh wave is shown in Figure 2a. When the LiNbO_3_/SU-8 is covered by an overcoat half-space (as shown in Figure 1b), the Rayleigh wave disappears, and a Stoneley wave propagates at a velocity larger than that of the Rayleigh wave: the IAW acoustic field is trapped in the LiNbO_3_ rather than distributed at the interface only for a specific choice of overcoat material and SU-8 layer thickness. Figure 2b and c show, as an example, the solid displacement of the IAW travelling in LiNbO_3_/SU-8/Al_2_O_3_ for two SU-8 layer thicknesses. The pictures demonstrate that an improved energy trapping toward the interface is obtained by increasing the SU-8 thickness; when the thickness of the SU-8 layer is further increased, the wave is guided inside the SU-8 layer. The pictures were obtained by using the same scale factor.

As it can be seen from Figure 2, the SU-8 layer thickness plays an important role in the design of the IAW-based device as it affects the distribution of the mechanical field, which can be concentrated at the LiNbO_3_ surface (as it is desirable) or distributed on both sides of the interface. Depending on the type of the selected piezoelectric substate, it may happen that the propagation of the IAWs is allowed only if an SU-8 layer is placed between the two half-spaces: this is not the case of ZX-LiTaO_3_/fused silica [9,10,11], but it is for the 128° YX-LiNbO_3_/overcoat. In the former case, the IAW travels even in the absence of the SU-8 layer, but a 5 µm-thick SU-8 layer is able to enhance the trapping of the wave energy toward the interface; in the latter case, the presence of the SU-8 layer is fundamental to excite the propagation of the low-loss IAW. The presence of the thin low-acoustic impedance SU-8 layer induces an energy trapping effect as that observed in waveguiding systems based on Love waves.

**Figure 3:**
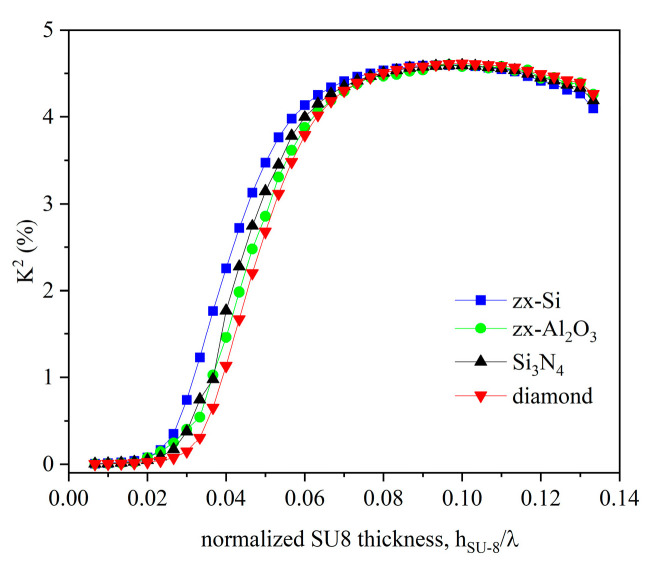
The K^2^ dispersion curves of the IAWs for Si, sapphire, diamond, and Si_3_N_4_ as overcoat materials atop the 128° YX-LiNbO_3_/SU-8 structure.

The IDT electrical-to-acoustic energy conversion efficiency was estimated by calculating the electromechanical coupling coefficient $K^{2}=2\cdot \frac{v_{free}-v_{ground}}{v_{free}}$, where *v_free_* and *v_ground_* are the wave velocity for electrically free and grounded boundary conditions onto the surface of the piezoelectric half-space. Figure 3 shows the K^2^ dispersion curves for different types of overcoat material and SU-8 layer thicknesses.

From Figure 3, it can be noticed that the K^2^ is close to zero for small SU-8 layer normalized thicknesses; with increasing the h_SU-8_ layer thickness, the K^2^ increases and reaches a quite high value (about 4.5%), which is a little lower than that (about 5.2%) of the SAW travelling along the surface of the bare 128° YX-LiNbO_3_. The shape of the K^2^ dispersion curve is similar for the four types of overcoat materials except for a limited h_SU8_⁄λ range (from 0.03 up to about 0.07): here, the Si offers the highest K^2^ while the isotropic diamond the lowest. For h_SU8_⁄λ > 0.08 and <0.13, the K^2^ dispersion curves merge and reach a plateau where the curves become undistinguishable and are not sensitive to the changes in the SU-8 layer thickness. Further increased h_SU8_⁄λ (>0.13) induces a decreased K^2^.

**Figure 4:**
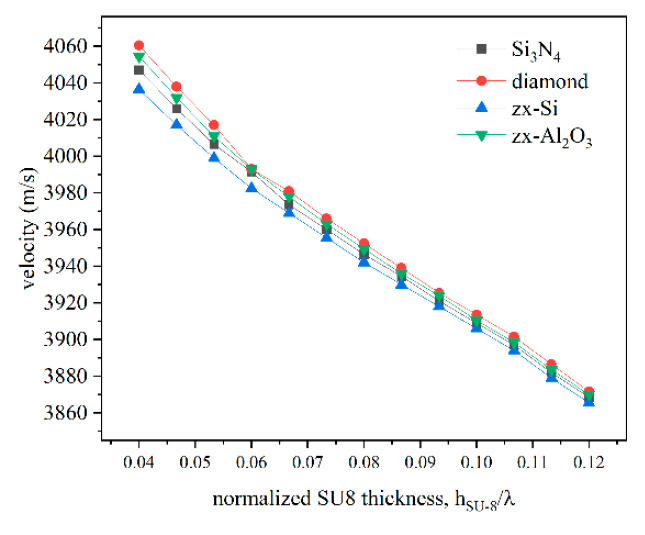
The frequency vs. the thickness of the SU-8 layer curves calculated for different overcoat material types.

Figure 4 shows the phase velocity of the IAW vs. the SU-8 layer thickness for different overcoat material types; the frequencies refer to the maximum of the admittance Y vs. frequency curves. Increasing SU-8 layer thicknesses correspond to a decreasing phase velocity of the IAW.

**Figure 5:**
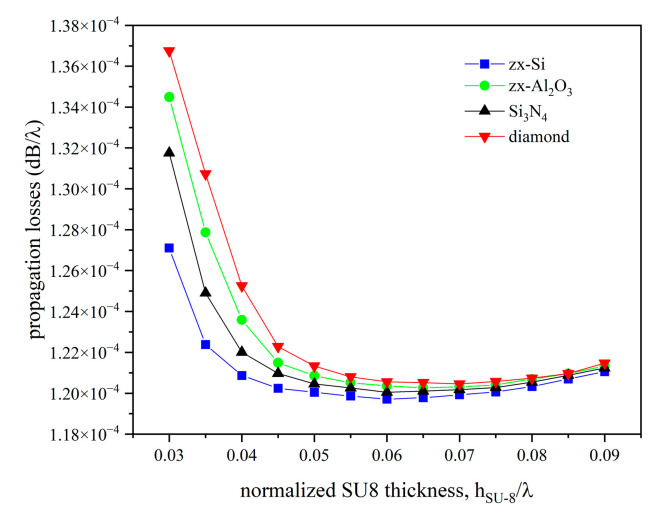
The propagation loss vs. the SU-8 layer thickness curves for different overcoat material types.

The wave propagation loss α is an important parameter that assesses the quality of the acoustic device performance: it is fundamental for the evaluation of the device performance as it refers to the reduction in signal as the IAW propagates along the interface of the two media. A major challenge is performing an optimized design of the device to minimize the propagation loss through a proper selection of the materials. At this aim, the admittance Y vs. the frequency curve was calculated by performing the frequency domain study of the LiNbO_3_/IDT/SU-8/overcoat structures. The IAW propagation loss α was estimated, for different types of overcoats and SU-8 layer thicknesses, as α=ω2Qvgr being vgr=vph·1+hSU−8λ∂vph∂hSU−8λ the group velocity, *v_ph_* = *λ*·*f_res_* the phase velocity, and ω = 2·π·*f_res_*; Q=Δffres was calculated from the Real(Y) vs. the frequency curve, where *f_res_* is the abscissa of the admittance peak, and ∆*f* is the peak bandwidth. Figure 5 shows the propagation loss vs. the SU-8 layer thickness curves assuming 0.0001 isotropic loss for all the materials.

The IAWs losses are high for thin as well as for thick SU-8 layers: in the former case, the propagation of IAWs is not allowed, while, in the latter case, the wave propagation is guided inside the SU-8 layer; the IAWs travel with low losses and high K^2^ for a limited range of SU-8 layer thicknesses. The data shown in Figure 5 are only indicative of the generic trend of α versus the thickness of the SU-8 layer since the structural losses attributed to each domain are generic. It is worth noting the correspondence between the curves of Figure 3 and Figure 5 where the increase of K^2^ and the reduction in losses are noted for the same thickness range.

**Figure 6:**
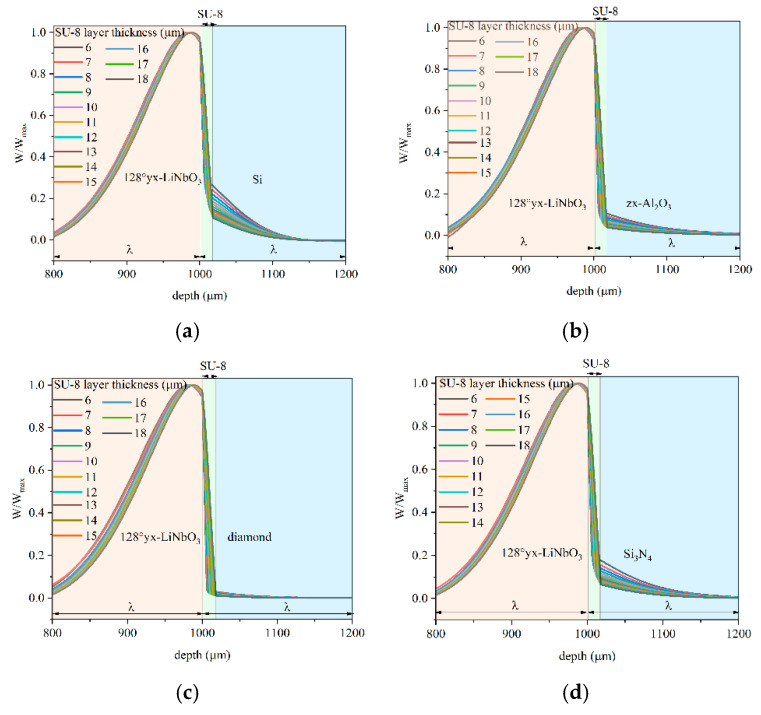
The w vs. depth curves at different SU-8 layer thicknesses and at λ = 200 µm for the (**a**) ZX-Silicon, (**b**) Z-sapphire, (**c**) diamond, and (**d**) Si_3_N_4_ overcoat.

Figure 6 shows the depth profile of the shear vertical displacement component *w* of the IAW along the whole structure: the w vs. depth curves were calculated at the resonant frequency corresponding to the maximum value of Real(Y), for different overcoat types and SU-8 layer thicknesses. The shear vertical displacement component *w* was estimated along the cut line positioned in the middle of one electrode, along the entire length of the unit cell; each curve was normalized to its maximum value (wwmax). The pictures show the *w* profile one wavelength below and above the LiNbO_3_ /SU-8 interface.

**Figure 7:**
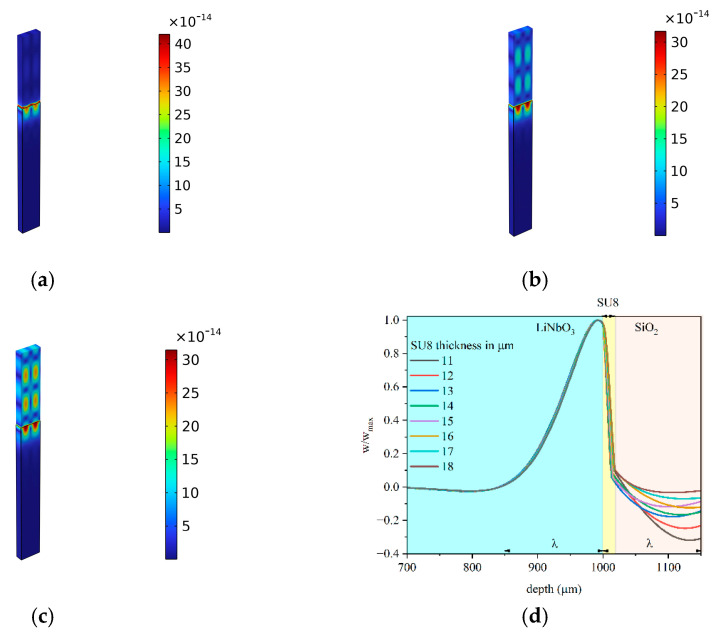
The solid displacement of the IAW travelling along LiNbO_3_/SU-8/SiO_2_, for different thicknesses of the SU-8 layer: (**a**) 18 µm, (**b**) 11 µm, and (**c)** 6 µm. (**d**) The w vs. depth curves at different SU-8 layer thicknesses for the SiO_2_ overcoat.

By comparing the pictures of Figure 6, it can be noticed that, with increasing the SU-8 layer thickness, the acoustic field is less concentrated in the overcoat but rather in the LiNbO_3_, especially when diamond or sapphire covers the SU-8 layer, and especially for small film thicknesses. For large SU-8 layer thicknesses, the *w* profiles quite merge. The best performance is obtained for the overcoat as fast as diamond and sapphire. In the case of an overcoat as slow as SiO_2_, the penetration of the acoustic field in the overcoat is large for thin SU-8 layer thicknesses and decreases for a large value: Figure 7 shows the solid displacement for some thicknesses of the SU-8 layer and the w vs. depth curves for a 150 µm wavelength.

From Figure 7, it can be noticed that the penetration of the interface wave into the overcoat increases for thin SU-8 layers; the mechanical displacement is mostly concentrated inside the piezoelectric half-space for a thicker SU-8 layer.

Overcoat materials with a Young modulus higher than that of the piezoelectric half-space allow the acoustic energy to be mostly concentrated in the LiNbO_3_ with a small leakage in the overcoat. Fast overcoat materials such as Si, diamond, and Al_2_O_3_ are very promising: Si is cheap, diamond is highly expensive, while Al_2_O_3_ is fast, transparent, and cheaper than diamond; the latter material seems the material of choice. The theoretically predicted propagation of the IAWs was experimentally assessed in LiNbO_3_/SU-8/glass, LiNbO_3_/SU-8/Si, and LiNbO_3_/SU-8/Al_2_O_3_ structures.

## 3. Experiment

### 3.1. Sample Preparation

**Figure 8:**
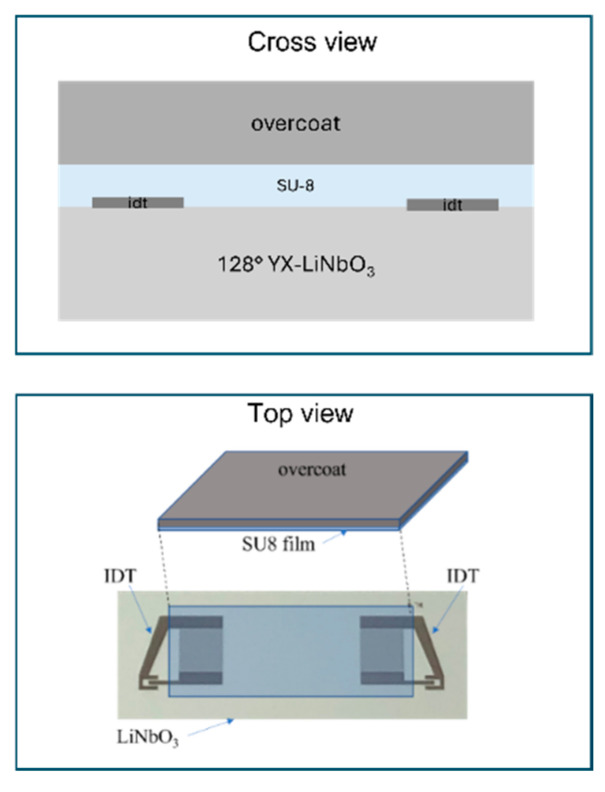
The schematic of the devices (cross and top views) fabricated to test the propagation of the IAWs (the picture is not in scale).

Based on the numerical results obtained by FEM studies, three different types of overcoat materials were used to fabricate the 128° YX-LiNbO_3_-based IAW structures. Firstly, a SAW delay line was fabricated onto a 128° YX-LiNbO_3_ bare substrate by a lift-off process and by following the technological procedure already described in other papers from the authors [15,16]. Two different types of delay line were fabricated: one type has an 80 µm wavelength in split finger configuration (number of finger pairs N = 80, IDTs centre-to-centre distance L = 6600 µm, fingers overlapping 1568 µm); the other has 200 µm wavelength in a single finger configuration (N = 18, L = 7750 µm, overlapping 8500 µm). The devices were mounted onto a printed circuit board (PCB) with an epoxy adhesive; the pads of the IDTs were electrically connected to the SMA connectors fixed onto the PCB by Al wires soldered by ultrasonic bonding. Finally, the overcoat was fixed atop the LiNbO_3_ substrate by applying a layer of SU-8 3025 negative epoxy photoresist from MicroChem Corporation. The SU-8 was spin-coated onto the overcoat (SiO_2_, Si and Al_2_O_3_) by applying the following procedure: 1. the SU-8 3025 bottle was cooled down for 30 min and the overcoat was dehydrated on a hotplate for 10 min at T = 200 °C; 2. the overcoat was cooled down, then 1 mL of SU-8 was dropped atop the overcoat and spun at 6000 rpm; 3. the overcoat/SU-8 substrate was positioned atop the LiNbO_3_/IDTs substrate and, finally, the whole structure was soft-baked in air at 95 °C for 10 min by means of a hotplate. Figure 8 shows the schematic of the devices (cross and top views) fabricated to test the propagation of the IAWs (the picture is not in scale).

We decided to place the SU-8/overcoat atop the entire delay line (including the two IDTs and the acoustic wave path) and not only in between the two IDTs to avoid the generation of spurious signals due to wave reflections, even if we are aware that our choice leads to a reduction in the electromechanical transduction efficiency of the IDTs covered by the SU-8/overcoat.

The thickness of the SU-8 layers spun at different rotational speeds was measured by making cross-sectional micrographs of the Si/SU-8/Si multilayers using a Zeiss Evo MA10 scanning electron microscope (SEM) (Carl Zeiss, Oberkochen, Germany). The SEM photos were taken after the Si/SU-8/Si wafers were diced on a Disco 321 Wafer Dicing Saw, with the dicing process considered as an adhesion test itself.

### 3.2. Device Test

The scattering parameter S_21_ of the IAW-based devices was measured in the frequency domain by using a vector network analyser (Keysight P9371A, Keysight Technologies, Inc., P9371A, Santa Rosa, CA, USA), which was connected to the PC for real-time acquisition; measures were performed in dark and controlled temperature and humidity. The measured S_21_ vs. frequency curves refer to untuned devices and were performed with the VNA calibrated (full two ports’ calibration) with a SOLT kit (SOLT = Short, Open, Load, Through) up to the coaxial cables. The calibration of the VNA excludes the test fixture, which consists of two SMA connectors mounted onto the PCB where the substrate is fixed. The curves were obtained by using time gating to cancel the spurious time signals and to obtain the idealized frequency response.

#### 3.2.1. SAW in Bare LiNbO_3_

**Figure 9:**
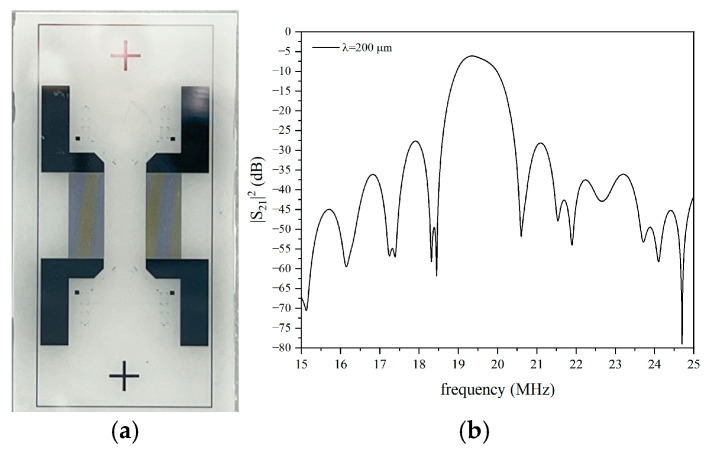
(**a**) The SAW delay line photo and (**b**) the S_21_ vs. frequency curve for the SAW delay line onto bare 128° YX-LiNbO_3_.

Figure 9a shows the photo of the SAW delay line with a wavelength of 200 µm fabricated onto the bare 128° YX-LiNbO_3_ substrate; Figure 9b shows the measured frequency responses of the device, i.e., the S_21_ vs. the frequency curve. The device’s operating frequency *f* is about 19.5 MHz (the SAW phase velocity *v_ph_* = *f*·*λ* is about 3900 m/s), while the losses are about 5 dB.

#### 3.2.2. IAW in LiNbO_3_/SU-8/SiO_2_

**Figure 10:**
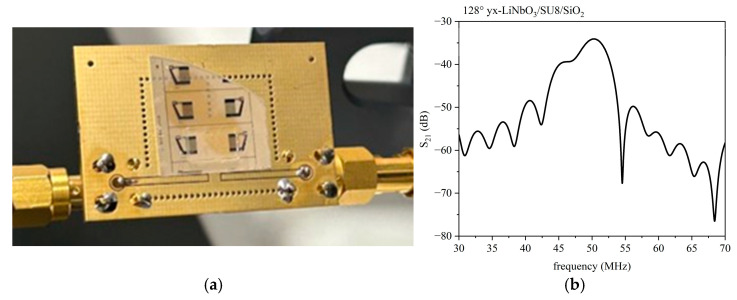
(**a**) The photo of the device; (**b**) The S_12_ vs. frequency curve for the LiNbO_3_/SU-8/SiO_2_. The delay line has λ = 80 µm.

Figure 10a shows the photo of the device fixed to the PCB: a transparent SiO_2_ overcoat (500 µm)/SU-8 covers the entire surface of the LiNbO_3_ substrate (500 µm thick), also including the two IDTs. The IDTs have λ = 80 µm and split finger configuration. Figure 10b shows the S_21_ vs. the frequency curve of the delay line with glass overcoat and SU-8 layer (about 15 µm thick).

The operating frequency of the tested device, evaluated at the peak of the S_21_ parameter vs. frequency curves, is equal to 50.21 MHz, and the loss is equal to 34.09.

#### 3.2.3. IAW in LiNbO_3_/SU-8/Si

**Figure 11:**
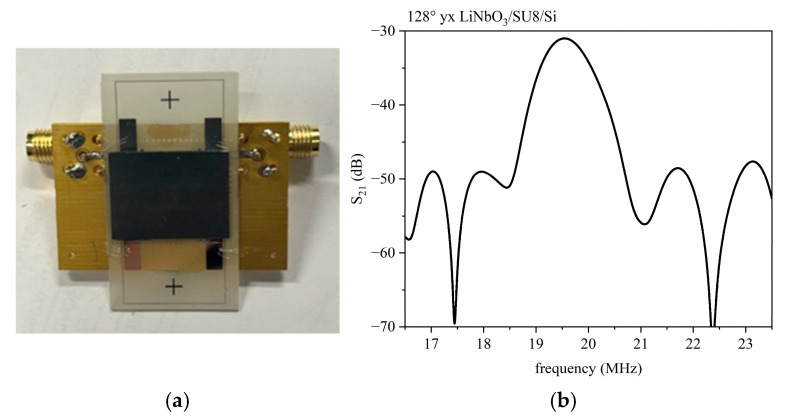
(**a**) The photo of the device; (**b**) The S_12_ vs. the frequency curve for the LiNbO_3_/SU-8/Si. The delay line has λ = 200 µm.

Figure 11a shows the photo of the LiNbO_3_/SU-8/Si-based device: the surplus SU-8 adhesive is deposited in the vicinity of the bonded area and clearly observable in the photo. Figure 11b shows the S_21_ vs. frequency curve of the delay line (λ = 200 µm) with the Si overcoat (500 µm thick) and SU-8 layer (about 15 µm thick); the Si overcoat covers the entire surface of the LiNbO_3_ (1 mm thick), also including the two IDTs. The IDTs have single finger configuration.

The operating frequency of the tested device, evaluated at the peak of the S_21_ parameter vs. frequency curves, is equal to 19.54 MHz, and the loss is equal to 31.0 dB.

#### 3.2.4. IAW in LiNbO_3_/SU-8/Sapphire

**Figure 12:**
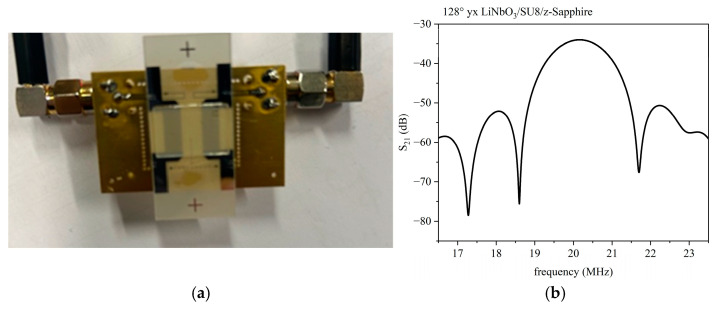
(**a**) The photo of the device; (**b**) The S_12_ vs. the frequency curve for the LiNbO_3_/SU-8/sapphire. The delay line has λ = 200 µm.

Figure 12a shows the photo of the LiNbO_3_/SU-8/c-sapphire-based device with a wavelength λ = 200 µm. The overcoat is single-side polished and has a thickness of 500 µm; it covers the entire surface of the delay line implemented onto the LiNbO_3_ substrate (1 mm thick). Figure 12b shows the S_21_ vs. the frequency curve of the delay line with a sapphire overcoat and an SU-8 layer (about 15 µm thick). The IDTs have single finger configuration.

The operating frequency of the tested device, evaluated at the peak of the S_21_ parameter vs. frequency curves, is equal to 20.16 MHz, and the loss is equal to 34.02 dB.

## 4. Discussion

The existence of the Stoneley wave propagating along a plane interface between two anisotropic media bonded through an SU-8 layer has been theoretically predicted and experimentally assessed. The experimental measures confirm that the IAW velocity is larger than that of the SAW travelling in the same half-space; moreover, the IAW insertion losses are larger than that of the SAW due to the unavoidable energy leakage in the SU-8 layer and overcoat. The theoretical results indicate that there are some optimized conditions for the SU-8 layer thickness and overcoat material type, which allow a Stoneley wave to be propagated with low loss, high K^2^, and a large shear vertical displacement component. Overcoat materials with a Young modulus higher than that of the piezoelectric half-space (such as Al_2_O_3_, Si, and diamond) ensure that the wave propagation is confined mainly in the LiNbO_3_, below the interface, although with minimal dispersion in the overcoat. The SU-8 layer plays a double role: it contributes to the adhesion of the two media and improves the IAW energy concentration at the interface. Table 2 lists the velocities of the longitudinal and of the two transverse bulk acoustic waves (LBAW, SHBAW1, and SHBAW2) and of the SAW of the materials presently studied: the velocities were calculated by using Comsol 6.1 software in the lossless-material approximation.

Fast overcoat materials with transverse BAW velocities larger than that of the 128° YX-LiNbO_3_ allow the propagation of IAWs even for small SU-8 layer thickness; in this sense, Si, diamond, and Al_2_O_3_ are very promising. On the contrary, SiO_2_ is slower than LiNbO_3_, but the presence of a thick SU-8 layer has the effect to slow down the LiNbO_3_ velocity and to allow an efficient propagation of IAWs.

The experimentally measured IAW velocity (about 4000 m/s) is quite similar for the three types of the tested devices and agrees with the values theoretically calculated. Also, the untuned loss at the operating frequency is quite similar for the three devices: these results confirm that the wave velocity is predominantly influenced by the LiNbO_3_ substrate and travels along its surface, which coincides with the interface of the two bulk media. As further confirmation of this, it is found that, when some drops of deionized water were placed on the free surface of the overcoat during the tests of the devices, the IAWs were not affected by the presence of water as it would instead happen in the case of SAWs. For a volume of water large enough to cover the entire surface of the device, it was observed that the wave remained unperturbed since the adhesion of the SU-8 layer to both the coating layer and the substrate was high enough to prevent the liquid from penetrating inside the structure. This confirms that the IAW energy is not irradiated towards the free surface, as demonstrated by the performed simulations. A further development of the LiNbO_3_/SU-8/overcoat system includes the subsequent fabrication of an embedded microfluidic channel in the overcoat, midway the two IDTs, by micromachining technique, to allow the coupling of the acoustic wave to the liquid flowing in the channel.

For all the structure compositions examined, the wave velocity is found to lie between the surface wave velocity of the LiNbO_3_ and the slowest transverse BAW velocity in the overcoat material. For the tested devices, it is demonstrated that the acoustic field is strong enough to allow performance competitive with that of SAW devices with PDMS walls. In Reference [10], it is clearly stated that typical SAW losses due to BAW radiation inside the PDMS walls and viscous losses are in the range 6–20 dB/cm.

Devices with quite thick SU-8 layers (more than 20 µm) were also fabricated at the very beginning of this research, but the S_21_ frequency responses were very noisy, and the IAW signal was hardly distinguishable. On the contrary, the peaks of the S_11_ and S_22_ scattering parameters vs. frequency curves were clearly visible although very attenuated.

## 5. Conclusions

This paper reports numerical simulations and experimental results on the propagation of Stoneley waves in the 128° YX-LiNbO_3_/SU-8/overcoat structures for different overcoat material types. The LiNbO_3_ substrate was chosen for its high K^2^ and large shear vertical displacement component *w* for SAW propagation: the former characteristic makes it suitable for the design of filters and resonators; the latter characteristic makes it the material of choice for microfluidic applications. The overcoat materials tested (Si, fused silica and sapphire) were chosen for their low cost and ease of being found. The SU-8 layer thickness value was chosen as to guarantee high K^2^ and large *w* for IAW propagation.

The 3D FEM study (eigenfrequency and frequency domain study) was performed to calculate the IAW characteristics (such as IAW phase velocity, propagation loss, K^2^ and displacement components) to obtain the design of the devices to fabricate and test. Since devices based on Si, diamond, and sapphire exhibit similar behaviour in terms of K^2^ and propagation loss, the Si seems the material of choice since it is cheaper than the others. Sapphire can be considered too for specific applications that take advantage of its optical transparency to combine acoustic and optical technologies to separate and distinguish cells with different optical properties [17].

The experimentally excited IAWs travel at velocity in agreement with the value theoretically predicted by 3D FEM analysis. The experimental measure confirmed that, by choosing the proper SU-8 layer thickness, the IAWs can be excited and revealed by the IDTs photolithographically implemented onto the piezoelectric LiNbO_3_ substrate. The ILs (about −30 dB) of our IAW-based devices are comparable to that reported in Reference [18], where the feasibility of a SAW device based on the 128° YX LiNbO_3_/PDMS channel is assessed for acoustophoretic application. The authors investigate the effects of the PDMS channel wall thickness on the insertion loss of the SAW travelling in 128° YX LiNbO_3_. They experimentally measure a 31.2% increase in the insertion loss (no liquid sample in the PDMS channel) for reducing the side wall thickness of the PDMS channel from 8 to 2 mm.

Although interest in Stoneley waves began a long time ago for packageless applications, today, it can be renewed for applications in the field of sensing and microfluidics. In the latter case, the conventional basic layout (consisting in the LiNbO_3_ substrate and PDMS channel) can be substituted by the combination of the LiNbO_3_ substrate and overcoat substrate, which undergoes the microchannel fabrication by means of dicing [19], laser ablation [20,21], or micromilling [22].

The measure of the structural losses of each material involved in the IAW-based device would help to improve the design of devices with a wide range of optimized characteristics such as thermal compensation, predominant polarization (such as shear vertical or shear horizontal), low loss, high frequency, and large K^2^.

The current results are proof of principles for the study of the configuration of a device based on the propagation of IAWs. The challenge in future work will be to find specific combinations of substrate and coating materials that ensure the propagation of low-loss IAWs; such optimized design can result in a highly efficient conversion of the IAW into a SAW inside the microchannel excavated in the overcoat. Future work may extend the design of this type of systems to also include unidirectional IDTs and different types of bonding layers [23] to reduce the wave propagation losses, or by using a different piezoelectric substrate and/or acoustoelectric coupling configuration.

## Figures and Tables

**Table 1:** Mass density, Young modulus, Poisson ratio, and elastic constants of the studied overcoat materials.

Overcoat Material	Mass Density (Kg/m^3^)	Young Modulus (GPa)	Poisson Ratio	Reference
SiO_2_	2200	70	0.17	Comsol library
Diamond	3515	1050	0.1	Comsol library
Si_3_N_4_	3100	250	0.23	Comsol library
SU-8	1190	4.02	0.22	[13]
		Elastic constants (GPa)		
Si (001)<100>	2330	c_11_ = 166; c_12_ = 64		Comsol library
Al_2_O_3_ (001)	3980	c_11_ = 497; c_12_ = 164; c_13_ = 111; c_14_ = −23.5; c_33_ = 498; c_44_ = 147; c_66_ = 166.5		[14]

**Table 2:** The BAW and SAW velocities of the studied materials.

Material/Orientation	LBAW (m/s)	SHBAW1 (m/s)	SHBAW2 (m/s)	SAW (m/s)
zx-Sapphire	11,174.72	6765.87	5743.86	5735.28
zx-Silicon	8440.65	5844.92	5844.92	4921.23
diamond	17,503.66	12,804.38	12,804.38	10,971.00
SiO_2_	5973.43	3765.88	3765.88	3411.15
Si_3_N_4_	8241.63	5266.48	5266.48	4756.16
128° YX-LiNbO_3_	6572.02	4794.77	4079.21	3994.80

## Data Availability

The original contributions presented in this study are included in the article. Further inquiries can be directed to the corresponding author.
